# Single-cell RNA-seq reveals vascular endothelial cell heterogeneity and potential vascular dysfunction in hypertrophic scars: scRNA-seq reveals endothelial cell heterogeneity in hypertrophic scars

**DOI:** 10.3724/abbs.2023001

**Published:** 2023-01-19

**Authors:** Renpeng Zhou, Angang Ding, Jialin Chen, Chen Wang, Yimin Liang

**Affiliations:** 1 Department of Plastic and Reconstructive Surgery Shanghai 9th People’s Hospital Shanghai Jiao Tong University School of Medicine Shanghai 200011 China; 2 Department of Ultrasonography Shanghai 9th People’s Hospital Shanghai Jiao Tong University School of Medicine Shanghai 200011 China

Hypertrophic scarring is a dermal fibroproliferative disorder characterized by excessive collagen secretion by fibroblasts. To date, most studies have mainly focused on the role of aberrant microRNAs, transcription factors and cytokines in regulating fibroblasts in hypertrophic scars [1]. A recent single-cell transcriptome analysis of hypertrophic scars also investigated alterations in fibrotic gene expression in fibroblasts [2]. However, increasing evidence has suggested the potential role of the microenvironment in the formation and progression of hypertrophic scars. Among them, vascular endothelial cells are recognized to be crucial for inflammation and fibrosis in pathological scars [3]. However, few studies have evaluated vascular endothelial cell profiling in hypertrophic scars. An in-depth scRNA-seq analysis of vascular endothelial cell heterogeneity in hypertrophic scars is necessary.

In the present study, we investigated endothelial cell heterogeneity within hypertrophic scars. First, we collected single-cell RNA sequencing matrices of normal skin and hypertrophic scars from published datasets and performed bioinformatics analysis (described in Supplementary data). Based on the scRNA-seq matrices, we identified 17 cell clusters ( Figure 1A). We assigned a cell type to each cluster according to the established lineage markers [4] ( Figure 1B, Supplementary Figure S1A and Supplementary Figure S1C). Vascular endothelial cells were represented by cluster 7, expressing PLVAP, ACKR1 and SELE; cluster 8, expressing SELE, ACKR1 and PLVAP; and cluster 10, expressing SELE. The percentage of vascular endothelial cells in total cells was higher in hypertrophic scar skin (16.3%) than in normal skin (8.34%, Supplementary Fig S1B).

**Figure 1:**
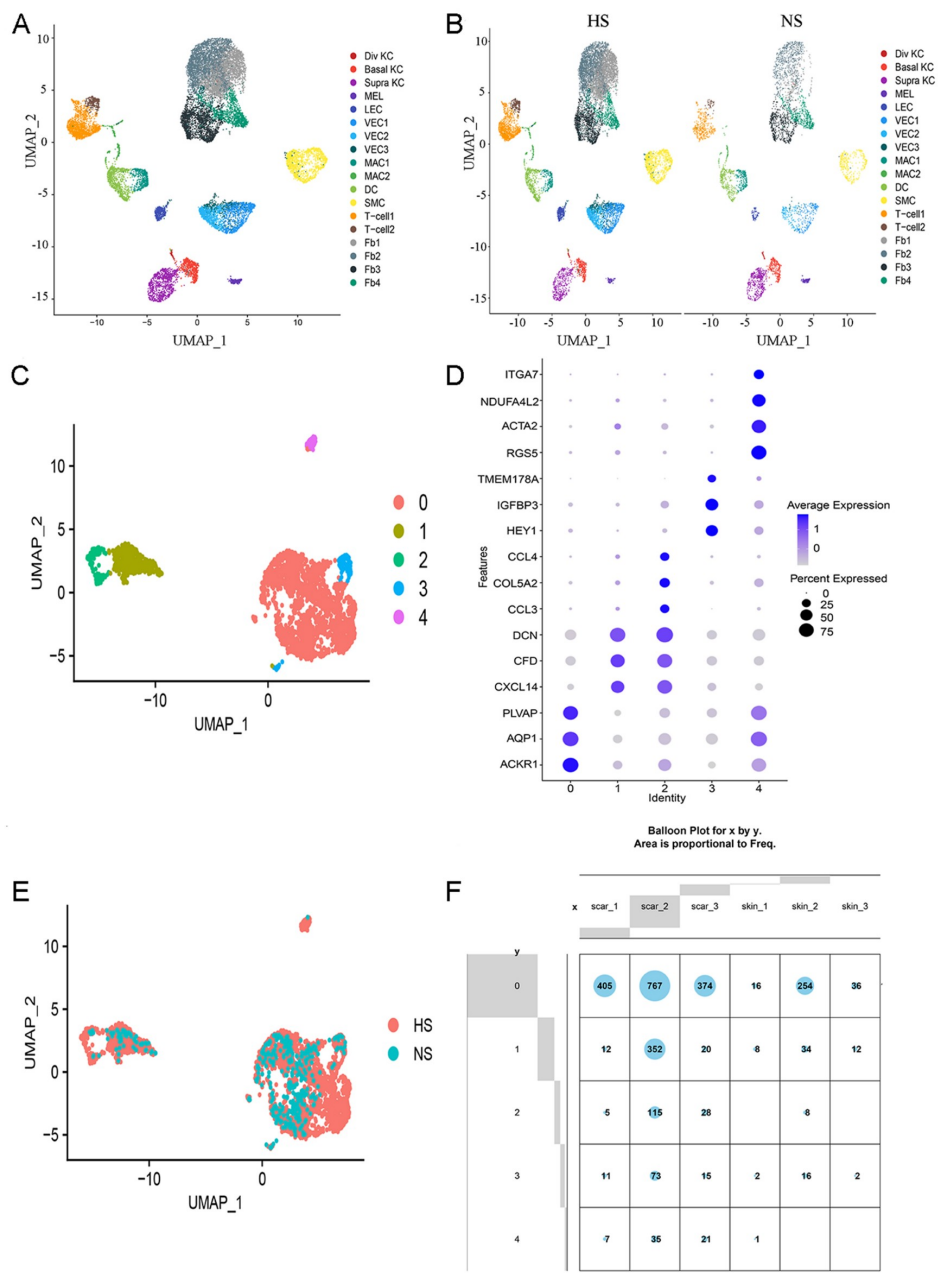
Figure 1 Identification of 17 individual cell populations and vascular endothelial cell heterogeneity in human hypertrophic scar UMAP visualization of 17 cell clusters in total samples using Seurat. (B) UMAP showing 17 cell clusters in human normal skin (NS) and hypertrophic scar (HS). (C) UMAP visualization of clustering revealing 5 distinct vascular endothelial cell populations. (D) Dot plot of cluster-identifying genes. (E) UMAP showing 5 subclusters of vascular endothelial cells in human normal skin (NS) and hypertrophic scar (HS). (F) The transcriptomes of 5 subclusters of vascular endothelial cells in each group.

Then, isolation and reclustering of vascular endothelial cell clusters revealed 5 distinct subclusters ( Figure 1C). Subcluster 0 presented the endothelial marker ACKR1, subclusters 1 and 2 presented the endothelial marker DCN, subcluster 3 presented the endothelial marker IGFBP3, and subcluster 4 expressed RGS5 and ACTA2 ( Figure 1D and Supplementary Figure S2A,B). ACKR1, which is restricted to small veins instead of arteries, capillaries and veins [5], was mostly expressed in subcluster 0. Thus, the largest component of vascular endothelial cells, subcluster 0, may represent small collecting veins. Subclusters 1 and 2 displayed DCN (decorin), representing microvessels or newly formed capillaries. Subcluster 3 expressed IGFBP3, representing endothelial precursor cells [6] ( Figure 1D and Supplementary Figure S2B). Subcluster 4 also expressed ACTA2 and RGS52, representing the large vessel and endothelial cells related to fibrosis. The UMAP (Uniform Manifold Approximation and Projection) and cell cluster data showed that subcluster 4 was mainly expressed in hypertrophic scars ( Figure 1E,F).

We further identified differentially expressed genes in vascular endothelial cells between the two groups. The data showed upregulated genes in vascular endothelial cells in hypertrophic scars, including the vascular markers NR2F2, FOXC1, VCAM1, VWF, ACTA2 and RGS5 ( Figure 2A and Supplementary Figure S3A). To validate the single-cell RNA-seq results, we stained the tissue sections with these differentially expressed genes using immunohistochemistry (described in Supplementary data). Staining of NR2F2, FOXC1, VCAM1, VWF, ACTA2 and RGS5 was more robust in hypertrophic scars than in normal skin ( Figure 2B and Supplementary Figure S3B). We also used qPCR to validate the expression levels of these genes in bulk tissues from normal skin and hypertrophic scar samples (described in Supplementary data). The mRNA expression levels of *Nr2f2*, *Vcam1*, *Acta2*, *Foxc1*, *Vwf* and *Rgs5* were upregulated in hypertrophic scars ( Supplementary Figure S3C), which confirmed that these genes were potential vascular markers for hypertrophic scars. Among them, FOXC1 is the molecular marker for arterial endothelial cells. NR2F2 (also known as COUP-TFII) is a known molecular marker of the venous endothelium [7]. Vascular cellular adhesion molecule-1 (VCAM-1) is an endothelial activation marker associated with inflammation as well as a cell adhesion molecule related to transendothelial migration of leukocytes such as macrophages [8].

**Figure 2:**
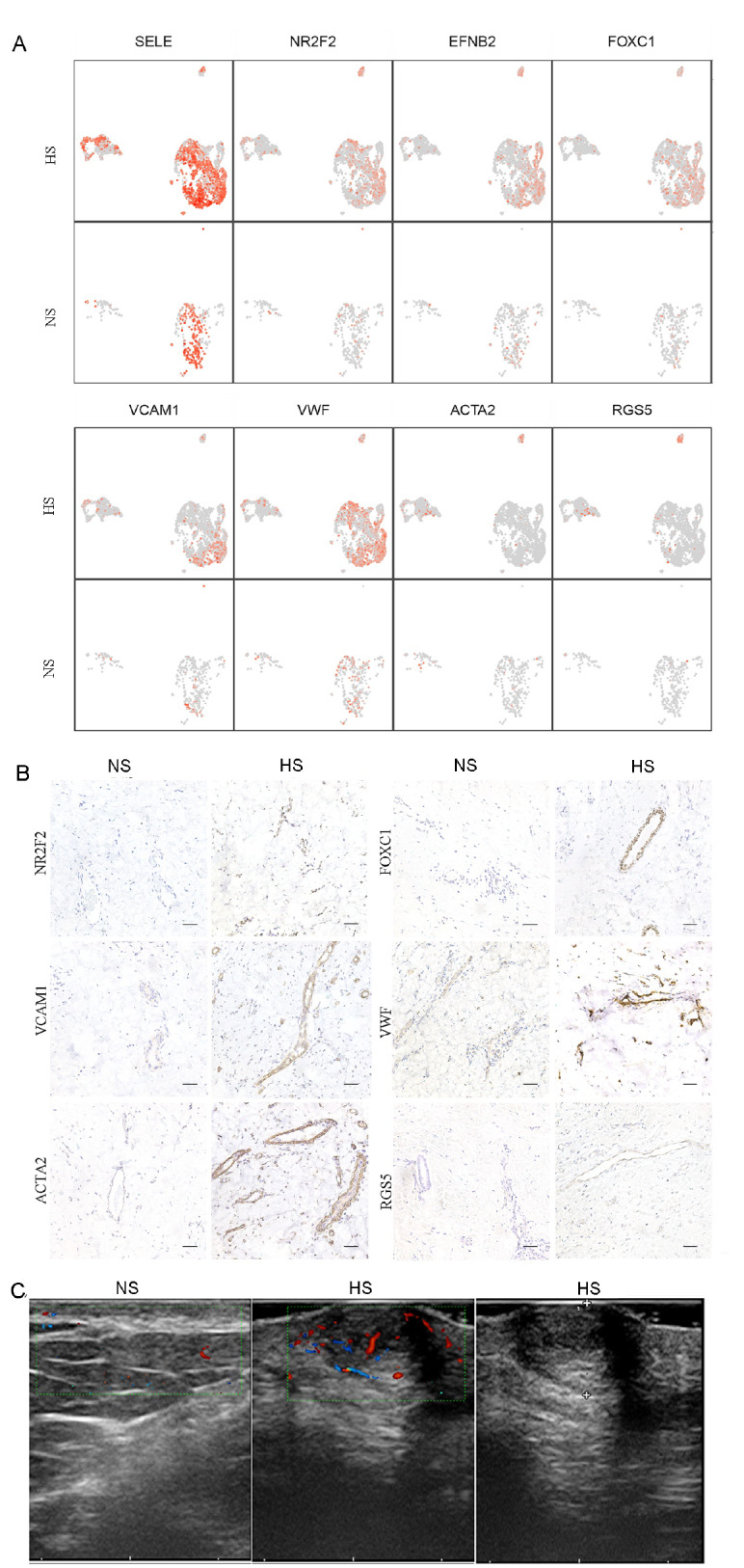
Figure 2 Upregulation and unique gene expression profile of vascular endothelial cells in human hypertrophic scars (A) UMAP plots showing representative differentially expressed genes of vascular endothelial cells between NS and HS. (B) IHC staining showing the expression of NR2F2, VCAM1, ACTA2, FOXC1, VWF and RGS5 in HS and NS. (C) Ultrasound showing higher vascularity in HS lesions than in NS lesions (left and medium panels). The right panel shows the thickness of the HS. Scale bar =50 μm.

Subcluster 4 expressed the typical pericyte marker RGS5 and the smooth muscle cell marker ACTA2. RGS5 is a pericyte marker that is consistently expressed in perivascular soft tissue tumors [9]. In human atherosclerotic plaques, the coexpression of endothelial markers and smooth muscle cells indicated endothelial to mesenchymal transition or mesenchymal to endothelial transition. Endothelial to mesenchymal transition promotes fibrosis in the wound vasculature [10]. In the present study, the unique expression of RGS5 and ACTA2 (subcluster 4) was observed in the vasculature of hypertrophic scars, which requires further experiments to clarify the effect of this cluster of endothelial cells on hypertrophic scars. Nevertheless, the single-cell RNA sequencing analysis of vascular endothelial cell heterogeneity in hypertrophic scars was based on public datasets, which need to be further investigated with our own single-cell sequencing data.

In addition, the hypertrophic scar and adjacent normal skin in patients were evaluated with ultrasound (described in Supplementary data). The ultrasound data showed significantly higher vascularity in hypertrophic scars than in adjacent normal skin ( Figure 2C and Supplementary Figure S3D), which indicated that the blood vessels in the lesion may be potential targets for treatment.

In summary, we identified 17 individual cell populations in human hypertrophic scars and revealed the differential gene expression profiles of vascular endothelial cells in hypertrophic scars and normal skin. We also verified the higher vascularity in hypertrophic scars and distinct markers of vascular endothelial cells. Our data provide an effective treatment targeting endothelial cells in hypertrophic scars.

## Supporting information

abbs-2022-391_supplementary

## Supplementary Data

Supplementary data are available at *Acta Biochimica et Biophysica Sinica* online.

## References

[1] Zhou R, Wang C, Lv D, Sun Y, Liang Y (2021). TNF-α inhibits fibrosis and migration of fibroblasts in hypertrophic scar by miR-141-3p. Acta Biochim Biophys Sin.

[2] Vorstandlechner V, Laggner M, Copic D, Klas K, Direder M, Chen Y, Golabi B (2021). The serine proteases dipeptidyl-peptidase 4 and urokinase are key molecules in human and mouse scar formation. Nat Commun.

[3] Ogawa R, Akaishi S (2016). Endothelial dysfunction may play a key role in keloid and hypertrophic scar pathogenesis – Keloids and hypertrophic scars may be vascular disorders. Med Hypotheses.

[4] Sun Y, Zhou R, Zhang H, Rong L, Zhou W, Liang Y, Li Q (2020). Skin is a potential host of SARS-CoV-2: a clinical, single-cell transcriptome-profiling and histologic study. J Am Acad Dermatol.

[5] Thiriot A, Perdomo C, Cheng G, Novitzky-Basso I, McArdle S, Kishimoto JK, Barreiro O (2017). Differential DARC/ACKR1 expression distinguishes venular from non-venular endothelial cells in murine tissues. BMC Biol.

[6] Chang KH, Chan-Ling T, McFarland EL, Afzal A, Pan H, Baxter LC, Shaw LC (2007). IGF binding protein-3 regulates hematopoietic stem cell and endothelial precursor cell function during vascular development. Proc Natl Acad Sci USA.

[7] Swift MR, Pham VN, Castranova D, Bell K, Poole RJ, Weinstein BM (2014). SoxF factors and Notch regulate nr2f2 gene expression during venous differentiation in zebrafish. Dev Biol.

[8] Kong DH, Kim Y, Kim M, Jang J, Lee S (2018). Emerging roles of vascular cell adhesion molecule-1 (VCAM-1) in immunological disorders and cancer. Int J Mol Sci.

[9] Shen J, Shrestha S, Yen YH, Scott MA, Soo C, Ting K, Peault B (2016). The pericyte antigen RGS5 in perivascular soft tissue tumors. Hum Pathol.

[10] Patel J, Baz B, Wong HY, Lee JS, Khosrotehrani K (2018). Accelerated endothelial to mesenchymal transition increased fibrosis via deleting notch signaling in wound vasculature. J Investig Dermatol.

