# Supplementary material for: Single-cell RNA-seq reveals vascular endothelial cell heterogeneity and potential vascular dysfunction in hypertrophic scars: scRNA-seq reveals endothelial cell heterogeneity in hypertrophic scars
Source: Acta Biochim Biophys Sin (Shanghai). 2023 Jan 19;55(1):165–8. doi: 10.3724/abbs.2023001 (PMC10157513; doi:10.3724/abbs.2023001)
Supplement: abbs-2022-391_supplementary [file abbs-2022-391_supplementary.pdf]

Supplementary information

Materials and Methods

Single-cell RNA-seq data pre-processing

The gene expression matrices for normal skin and hypertrophic scar (n=3) were downloaded from GEO database (GSE156326). The matrices were processed by Seurat (version 4.0.5) R package. Low quality cells with less than 300 expressed genes and 20% mitochondrial genes were excluded. Genes expressed in less than three cells were also removed. The doublets were detected with DoubleFinder 2.0.3 and removed.

Dimensionality reduction and clustering

The generated variable genes were used to perform principle component analysis (PCA). We then used the first 20 principle components (PCs) to construct a two-dimensional representation of the data using Uniform manifold approximation and projection (UMAP) with perplexity 10. This representation was then used to visualize the data.

Clusters were identified from PCA-reduced expression data at a resolution of 0.8 using the Seurat "FindClusters" algorithm, which calculates the neighborhood overlap between every cell and its nearest neighbors. Graph-based clustering results were visualized in 2-dimension using UMAP. Individual samples and sample groups were also visualized using UMAP. We annotated all cell clusters based on our previous established lineage markers [1].

Subclustering of the major cell types

To identify sub-clusters within endothelial cell types, we first get annotated clusters form cells form raw Seurat object using the “SubsetData” function of the Seurat package. Dimensionality reduction was performed using PCA in each cell type on variable genes as described above. Using the graph-based clustering approach implemented in the “FindClusters” function of the Seurat package, with a conservative resolution of 0.1 and otherwise default parameters, each cell type was reclustered by its principle components. For visualization purposes, these informative principle components were converted into UMAP plots as above.

Identification of cluster marker genes and differential expression analysis

The cluster-specific marker genes were identified using differential expression analysis. The difference between clusters was analyzed by “FindAllMarkers” function in the Seurat package. A marker gene was identified when it was expressed in a minimum of 25% of cells and at a minimum log fold change threshold of 0.25. In paired analyses, the log fold change and adjusted *P* value of each differentially expressed gene were calculated by using the non-parametric two-sided Wilcoxon rank-sum test and *P*-value less than 0.05 were considered to be significant.

Patients and tissues

For Peer Review

Normal skin tissues (*n*=8) and hypertrophic scar tissues (*n*=8) were harvested between Oct 2019 and Nov 2021 at Shanghai Ninth Hospital (Shanghai, China). The study was approved by the Institutional Review Board of Shanghai Ninth Hospital. Written informed consent was obtained from each participant.

Ultrasound evaluations of hypertrophic scars

Hypertrophic scars and adjacent normal skin were evaluated with high-resolution ultrasound with 22-MHZ probe (Esaote Mylab) for the assessment of scar thickness and vascularity in 20 patients. The red and blue color indicated the blood direction contrast to probe instead of artery or vein.

Immunohistochemistry staining

The skin tissues were fixed in 4% formalin and embedded in paraffin. The paraffin-embedded sections were subjected to immunohistochemistry. Briefly, the sections were deparaffinized and subject to heat-induced antigen retrieval and permeabilization. Subsequently, sections were incubated with 10% normal donkey serum and then incubated with primary antibodies at 4°C overnight. The primary antibodies were as follows: rabbit-anti-CD62E (SELE, ab185698, Abcam,Cambridge, UK), rabbit-anti-NR2F2 (ab211777), rabbit-anti-EFNB2 (ab131536, Abcam), rabbit-anti-FOXC1 (ab227977, Abcam), rabbit-anit-VCAM1 (ab134047, Abcam), rabbit-anti-VWF (ab179451, Abcam), mouse-anit-ACTA2 (ab7817, Abcam), rabbit-anti-RGS5 (ab196799, Abcam), and rabbit-anti-HIF-1alpha (ab51608, Abcam). The sections were sequentially washed with 0.5% PBST and incubated with secondary antibody, followed by staining with DAB kit. Finally, the slides were stained with hematoxylin. The staining was imaged under a microscope (Olympus, Tyoko, Japan). The Image-Pro Plus was used for the grayscale analysis of the positively stained cells.

Quantitative polymerase chain reaction (qPCR) analysis

Total RNA was extracted from normal skin tissues and hypertrophic scar tissues using TRIzol reagent (Invitrogen, Carlsbad, USA). Total RNA was reverse transcribed into cDNA using a reverse transcription kit (Taraka, Dalian, China) according to the manufacturer's instruction. qPCR was conducted with SYBR Green Master Mix (Taraka). The ΔΔCt method was used for quantifying gene expression of *Nr2f2*, *Vcam1*, *Acta2*, *Foxc1*, *Vwf* and *RGs5* relative to GAPDH. The experiments were done in triplicate. The primers were as follows: *Nr2f2* F: 5′-GCCATAGTCCTGTTCACCT-3′; R: 5′-GCACACTGAGACTTTTCCTG-3′; *Vcam1* F: 5′-CGAATGAGGGGACCACATCTA-3′; R: 5′-TGTTCGTTCCCAAAACTAACAGG-3′; *Acta2* F: 5′-AGCCAAGCACTGTCAGGAAT-3′; R: 5′-CACCATCACCCCCTGATGTC-3′; *Foxc1* F: 5′-TCACAGAGGATCGGCTTGAAC-3′; R:5′-TCCTGCTTTGGGGTTCGATT-3′; *Vwf* F: 5′-TAAGTCTGAAGTAGAGGTGG-3′; R: 5′-AGAGCAGCAGGAGCACTGGT-3′; *Rgs5* F: 5′-CCACCTGCCAAAATGTGCAA-3′; R:

For Peer Review

5′-GGCTGGTTTCTCTGGCTTCT-3′; *Gapdh* F: 5′-GACCCCTTCATTGACCTCAAC-3′; R: 5′-CTTCTCCATGGTGGTGAAGA-3′.

Statistical analysis

Data are presented as the mean ± standard deviation. Student's *t*-test was performed for comparison between two groups. For single-cell RNA sequence, differentially expressed genes were calculated by using the non-parametric two-sided Wilcoxon rank-sum test.

References

1. Sun Y, Zhou R, Zhang H, Rong L, Zhou W, Liang Y, Li Q. Skin is a potential host of SARS-CoV-2: A clinical, single-cell transcriptome-profiling and histologic study. J Am Acad Dermatol. 2020 Dec;83(6):1755-1757.

For Peer Review

Supplemental Figures

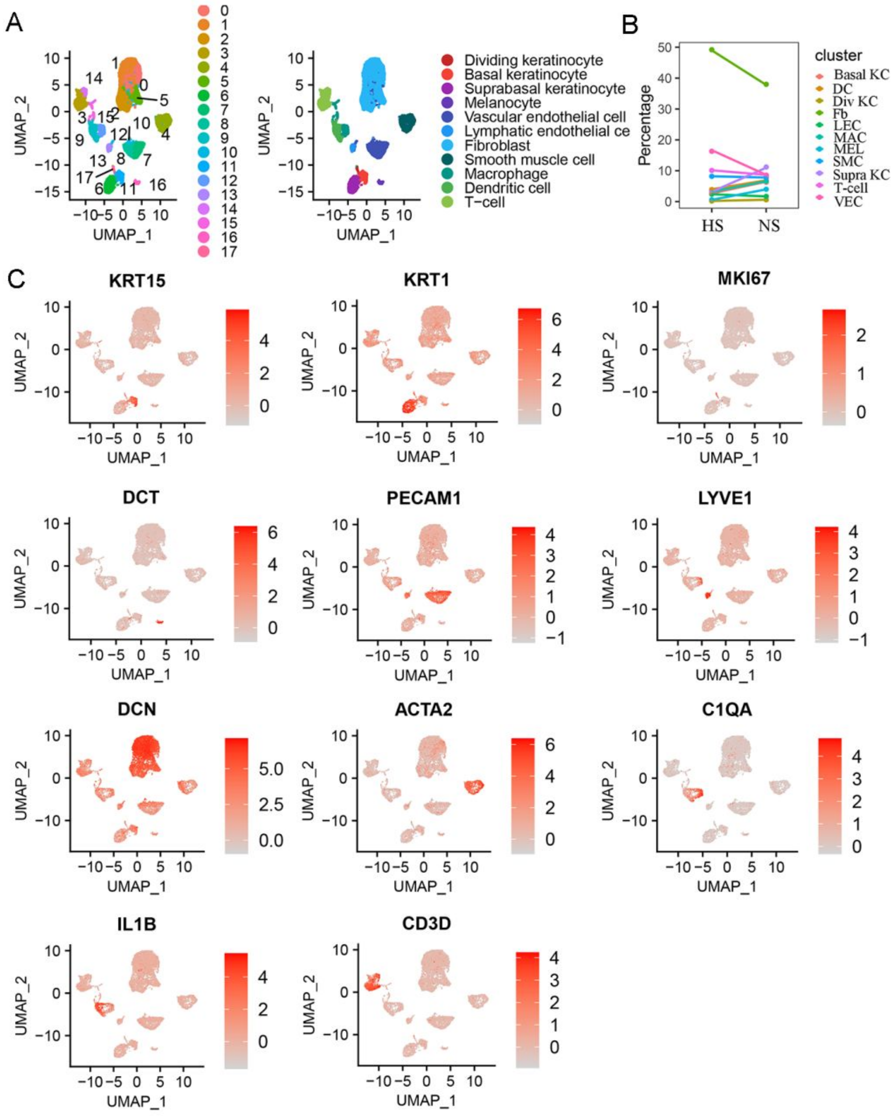

Supplementary Figure S1. Cell type identification by scRNA-seq analysis in hypertrohphic scars (A) Unbiased clustering of 18,411 cells reveals 17 cellular clusters (left) and 11 cell types (right). (B) The proportion of cell lineages in hypertrophic scars (HS) and normal skin (NS). (C) Feature plots of expression distribution for selected cluster-specific genes.

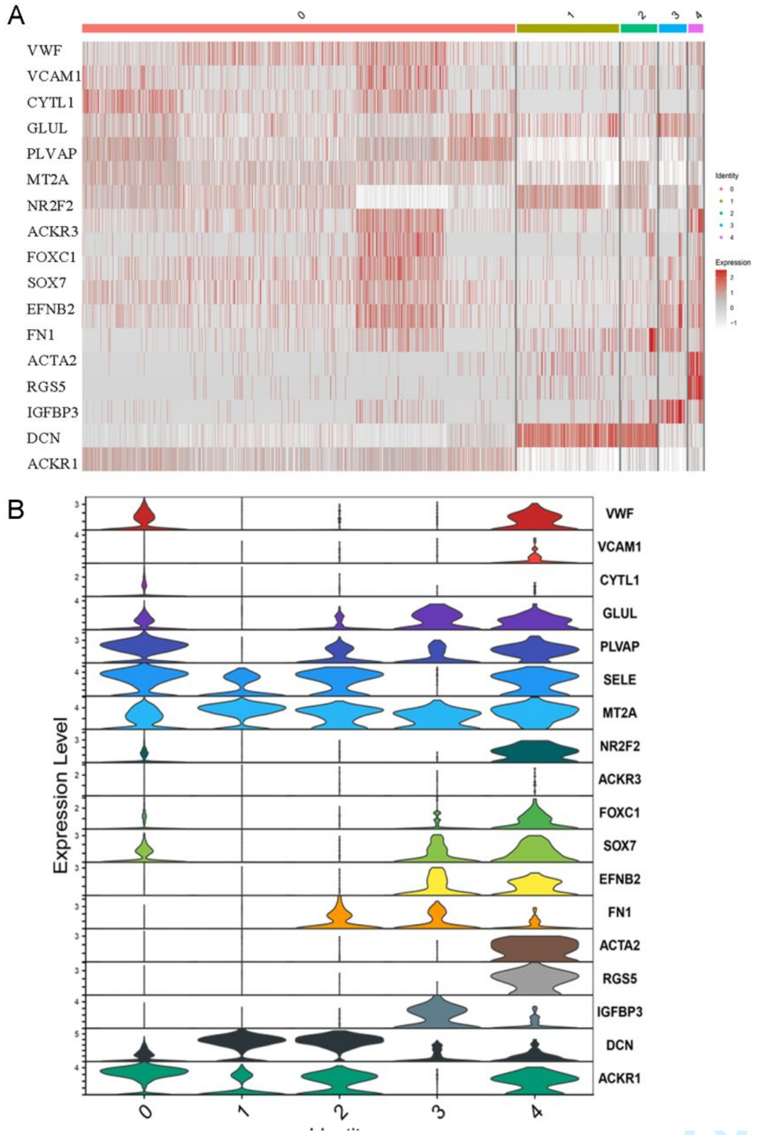

Supplementary Figure S2. Sub-cluster identification of vascular endothelial cells
(A) Heatmap showing the differentially expressed genes for each sub-cluster of vascular endothelial cells. (B) Violin plots showing representative differentially expressed genes in each sub-cluster of vascular endothelial cells.

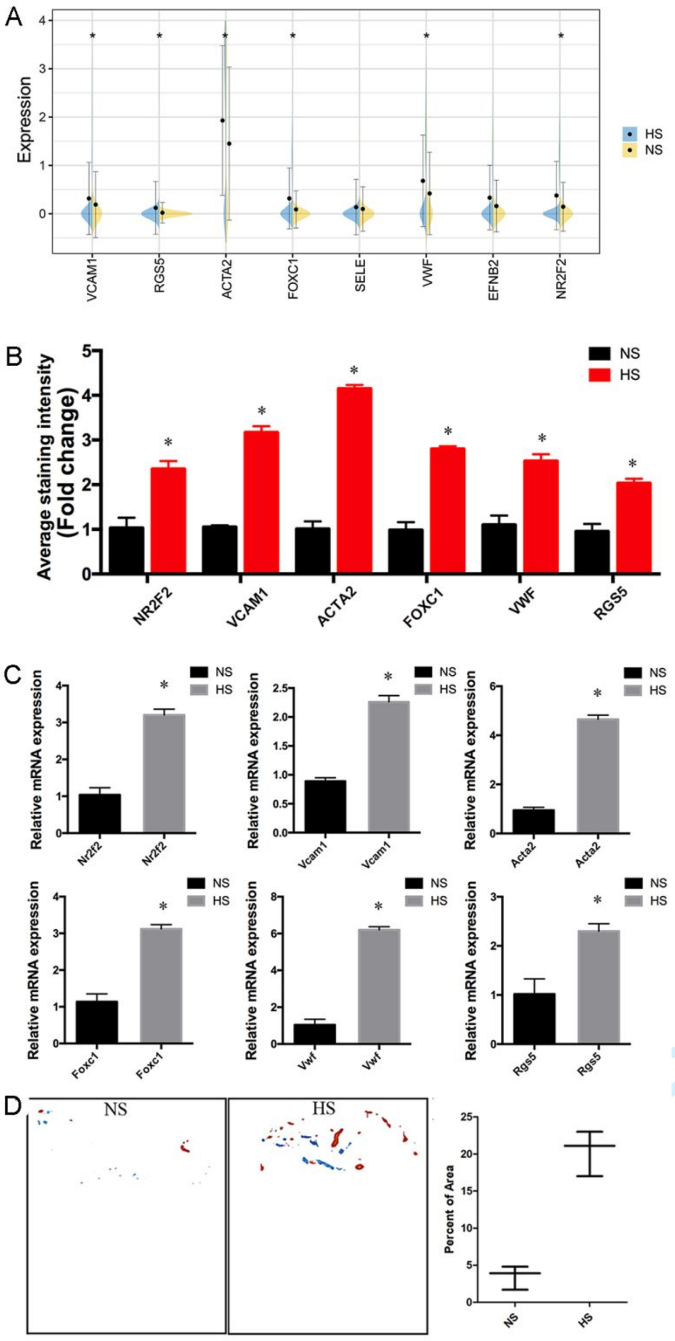

Supplementary Figure S3. Changes in the gene expression of vascular endothelial cells in hypertrophic scars (A) Violin plots showing differentially expressed genes of vascular endothelial cell between hypertrophic scars and normal skin. (B) Quantitative analysis of immunohistochemistry of expression of NR2F2, VCAM1, ACTA2, FOXC1,VWF and RGS5 in NS and HS. (C) Increased mRNA levels of Nr2f2, Vcam1, Acta2, Foxc1, Vwf and Rgs5 in bulk HS tissues. (D) The quantification of vascularity in HS and NS. NS: normal skin, HS: hypertrophic scars. *$P < 0.05$.
